# Supplementary material for: A Multi-Modal Dataset for Ground Reaction Force Estimation Using Consumer Wearable Sensors
Source: Sci Data. 2026 Apr 10;13:855. doi: 10.1038/s41597-026-07183-6 (PMC13243636; doi:10.1038/s41597-026-07183-6)
Supplement: Supplementary file 1 — Supplementary information [file 41597_2026_7183_MOESM1_ESM.pdf]

Speed measurements template (timing gates)

participant	activity	trial_num	gate_distance_m	time_s	speed_mps	speed_kmh
P1	walking	1	373	252	148	53
P1	walking	2	373	215	173	62
P1	walking	3	373	226	165	59
P1	walking	4	373	2	187	67
P1	walking	5	373	212	176	63
P1	walking	6	373	218	171	62
P1	walking	7	373	2	187	67
P1	walking	8	373	22	170	61
P1	walking	9	373	233	160	58
P1	walking	10	373	208	179	65
P1	jogging	1	373	113	330	119
P1	jogging	2	373	107	349	125
P1	jogging	3	373	099	377	136
P1	jogging	4	373	107	349	125
P1	jogging	5	373	122	306	110
P1	jogging	6	373	103	362	130
P1	jogging	7	373	106	352	127
P1	jogging	8	373	13	287	103
P1	jogging	9	373	121	308	111
P1	jogging	10	373	12	311	112
P1	jogging	11	373	11	339	122
P1	running	1	373	1	373	134
P1	running	2	373	092	405	146
P1	running	3	373	092	405	146
P1	running	4	373	069	541	195
P1	running	5	373	087	429	154
P1	running	6	373	089	419	151
P1	running	7	373	093	401	144
P1	running	8	373	086	434	156
P1	running	9	373	089	419	151
P1	running	10	373	091	410	148
P1	running	11	373	09	414	149
P1	running	12	373	084	444	160
P2	walking	1	395	238	166	60
P2	walking	2	395	255	155	56
P2	walking	3	395	254	156	56
P2	walking	4	395	263	150	54
P2	walking	5	395	263	150	54
P2	walking	6	395	248	159	57
P2	walking	7	395	248	159	57
P2	walking	8	395	246	161	58
P2	walking	9	395	243	163	59
P2	walking	10	395	253	156	56

P2	jogging	1	395	234	169	61
P2	jogging	2	395	177	223	80
P2	jogging	3	395	162	244	88
P2	jogging	4	395	154	256	92
P2	jogging	5	395	152	260	94
P2	jogging	6	395	156	253	91
P2	jogging	7	395	161	245	88
P2	jogging	8	395	154	256	92
P2	jogging	9	395	153	258	93
P2	jogging	10	395	151	262	94
P2	running	1	395	125	316	114
P2	running	2	395	133	297	107
P2	running	3	395	125	316	114
P2	running	4	395	129	306	110
P2	running	5	395	138	286	103
P2	running	6	395	139	284	102
P2	running	7	395	131	302	109
P2	running	8	395	133	297	107
P2	running	9	395	137	288	104
P2	running	10	395	136	290	105
P3	walking	1	373	327	114	41
P3	walking	2	373	280	133	48
P3	walking	3	373	295	126	46
P3	walking	4	373	286	130	47
P3	walking	5	373	290	129	46
P3	walking	6	373	281	133	48
P3	walking	7	373	290	129	46
P3	walking	8	373	272	137	49
P3	walking	9	373	277	135	48
P3	walking	10	373	281	133	48
P3	jogging	1	373	175	213	77
P3	jogging	2	373	163	229	82
P3	jogging	3	373	174	214	77
P3	jogging	4	373	188	198	71
P3	jogging	5	373	171	218	79
P3	jogging	6	373	179	208	75
P3	jogging	7	373	172	217	78
P3	jogging	8	373	184	203	73
P3	jogging	9	373	188	198	71
P3	jogging	10	373	168	222	80
P3	jogging	11	373	118	316	114
P3	running	1	373	125	298	107
P3	running	2	373	132	283	102
P3	running	3	373	134	278	100
P3	running	4	373	130	287	103
P3	running	5	373	130	287	103
P3	running	6	373	118	316	114
P3	running	7	373	127	294	106

P3	running	8	373	125	298	107
P3	running	9	373	136	274	99
P3	running	10	373	123	303	109
P4	walking	1	395	226	175	63
P4	walking	2	395	260	152	55
P4	walking	3	395	245	161	58
P4	walking	4	395	240	165	59
P4	walking	5	395	258	153	55
P4	walking	6	395	262	151	54
P4	walking	7	395	240	165	59
P4	walking	8	395	256	154	56
P4	walking	9	395	256	154	56
P4	walking	10	395	245	161	58
P4	walking	11	395	260	152	55
P4	jogging	1	395	155	255	92
P4	jogging	2	395	149	265	95
P4	jogging	3	395	149	265	95
P4	jogging	4	395	148	267	96
P4	jogging	5	395	135	293	105
P4	jogging	6	395	138	286	103
P4	jogging	7	395	141	280	101
P4	jogging	8	395	142	278	100
P4	jogging	9	395	153	258	93
P4	jogging	10	395	147	269	97
P4	running	1	395	116	341	123
P4	running	2	395	116	341	123
P4	running	3	395	121	326	118
P4	running	4	395	128	309	111
P4	running	5	395	130	304	109
P4	running	6	395	120	329	119
P4	running	7	395	130	304	109
P4	running	8	395	128	309	111
P4	running	9	395	120	329	119
P4	running	9	395	117	338	122
P5	walking	9	395	261	151	54
P5	walking	9	373	244	153	55
P5	walking	9	373	263	142	51
P5	walking	9	373	247	151	54
P5	walking	9	373	246	152	55
P5	walking	9	373	243	153	55
P5	walking	9	373	242	154	55
P5	walking	9	373	251	149	53
P5	walking	9	373	240	155	56
P5	walking	9	373	243	153	55
P5	walking	9	373	243	153	55
P5	jogging	1	373	133	280	101
P5	jogging	2	373	135	276	99
P5	jogging	3	373	128	291	105

P5	jogging	4	373	140	266	96
P5	jogging	5	373	132	283	102
P5	jogging	6	373	130	287	103
P5	jogging	7	373	129	289	104
P5	jogging	8	373	131	285	103
P5	jogging	9	373	128	291	105
P5	jogging	10	373	135	276	99
P5	running	1	373	111	336	121
P5	running	2	373	100	373	134
P5	running	3	373	114	327	118
P5	running	4	373	111	336	121
P5	running	5	373	104	359	129
P5	running	6	373	092	405	146
P5	running	7	373	098	381	137
P5	running	8	373	095	393	141
P5	running	9	373	104	359	129
P5	running	10	373	100	373	134
P5	running	11	373	096	389	140
P6	walking	1	395	276	143	52
P6	walking	2	395	330	120	43
P6	walking	3	395	273	145	52
P6	walking	4	395	326	121	44
P6	walking	5	395	303	130	47
P6	walking	6	395	300	132	47
P6	walking	7	395	329	120	43
P6	walking	8	395	271	146	52
P6	walking	9	395	337	117	42
P6	walking	10	395	325	122	44
P6	jogging	1	395	158	250	90
P6	jogging	2	395	166	238	86
P6	jogging	3	395	150	263	95
P6	jogging	4	395	171	231	83
P6	jogging	5	395	168	235	85
P6	jogging	6	395	162	244	88
P6	jogging	7	395	172	230	83
P6	jogging	8	395	162	244	88
P6	jogging	9	395	176	224	81
P6	jogging	10	395	169	234	84
P6	running	1	395	133	297	107
P6	running	2	395	133	297	107
P6	running	3	395	136	290	105
P6	running	4	395	130	304	109
P6	running	5	395	130	304	109
P6	running	6	395	131	302	109
P6	running	7	395	130	304	109
P6	running	8	395	130	304	109
P6	running	9	395	126	313	113
P6	running	10	395	126	313	113

P7	walking	1	395	173	228	82
P7	walking	2	395	284	139	50
P7	walking	3	395	278	142	51
P7	walking	4	395	283	140	50
P7	walking	5	395	281	141	51
P7	walking	6	395	281	141	51
P7	walking	7	395	307	129	46
P7	walking	8	395	286	138	50
P7	walking	9	395	278	142	51
P7	walking	10	395	283	140	50
P7	walking	11	395	303	130	47
P7	walking	12	395	302	131	47
P7	walking	13	395	282	140	50
P7	walking	14	395	294	134	48
P7	walking	15	395	292	135	49
P7	walking	16	395	302	131	47
P7	jogging	1	395	196	202	73
P7	jogging	2	395	196	202	73
P7	jogging	3	395	203	195	70
P7	jogging	4	395	190	208	75
P7	jogging	5	395	190	208	75
P7	jogging	6	395	177	223	80
P7	jogging	7	395	197	201	72
P7	jogging	8	395	180	219	79
P7	jogging	9	395	164	241	87
P7	jogging	10	395	150	263	95
P7	jogging	11	395	175	226	81
P7	jogging	12	395	164	241	87
P7	running	1	395	150	263	95
P7	running	2	395	167	237	85
P7	running	3	395	173	228	82
P7	running	4	395	166	238	86
P7	running	5	395	162	244	88
P7	running	6	395	168	235	85
P7	running	7	395	160	247	89
P7	running	8	395	160	247	89
P7	running	9	395	158	250	90
P7	running	10	395	160	247	89
P7	running	11	395	162	244	88
P8	walking	1	395	256	154	56
P8	walking	2	395	267	148	53
P8	walking	3	395	276	143	52
P8	walking	4	395	278	142	51
P8	walking	5	395	276	143	52
P8	walking	6	395	278	142	51
P8	walking	7	395	276	143	52
P8	walking	8	395	286	138	5
P8	walking	9	395	286	138	5

P8	walking	10	395	252	157	56
P8	jogging	1	395	153	258	93
P8	jogging	2	395	149	265	95
P8	jogging	3	395	14	282	102
P8	jogging	4	395	146	271	97
P8	jogging	5	395	137	288	104
P8	jogging	6	395	137	288	104
P8	jogging	7	395	138	286	103
P8	jogging	8	395	137	288	104
P8	jogging	9	395	142	278	10
P8	jogging	10	395	137	288	104
P8	jogging	11	395	135	293	105
P8	running	1	395	109	362	13
P8	running	2	395	101	391	141
P8	running	3	395	109	362	13
P8	running	4	395	103	383	138
P8	running	5	395	102	387	139
P8	running	6	395	118	335	121
P8	running	7	395	117	338	122
P9	walking	1	373	278	134	48
P9	walking	2	373	305	122	44
P9	walking	3	373	275	136	49
P9	walking	4	373	270	138	50
P9	walking	5	373	297	126	45
P9	walking	6	373	276	135	49
P9	jogging	1	373	196	190	69
P9	jogging	2	373	183	204	73
P9	jogging	3	373	184	203	73
P9	jogging	4	373	187	199	72
P9	jogging	5	373	192	194	70
P9	jogging	6	373	201	186	67
P9	running	2	373	135	276	99
P9	running	3	373	143	261	94
P9	running	4	373	134	278	100
P9	running	5	373	142	263	95
P9	running	6	373	146	255	92
P9	running	7	373	157	238	86
P10	walking	2	373	262	142	51
P10	walking	3	373	280	133	48
P10	walking	4	373	280	133	48
P10	walking	5	373	243	153	55
P10	walking	6	373	174	214	77
P10	walking	8	373	230	162	58
P10	walking	9	373	242	154	55
P10	walking	10	373	242	154	55
P10	walking	11	373	237	157	57
P10	jogging	1	373	131	285	103
P10	jogging	2	373	138	270	97

P10	jogging	3	373	135	276	99
P10	jogging	4	373	130	287	103
P10	jogging	5	373	135	276	99
P10	jogging	6	373	131	285	103
P10	jogging	7	373	131	285	103
P10	jogging	8	373	134	278	100
P10	jogging	9	373	134	278	100
P10	jogging	10	373	132	283	102
P10	jogging	11	373	122	306	110
P10	running	1	373	124	301	108
P10	running	2	373	131	285	103
P10	running	3	373	127	294	106
P10	running	4	373	122	306	110
P10	running	5	373	131	285	103
P10	running	6	373	125	298	107
P10	running	7	373	121	308	111
P10	running	8	373	121	308	111
P10	running	9	373	120	311	112
P10	running	10	373	119	313	113
P10	running	11	373	113	330	119
