# Supplementary material for: A Multi-Modal Dataset for Ground Reaction Force Estimation Using Consumer Wearable Sensors
Source: Sci Data. 2026 Apr 10;13:855. doi: 10.1038/s41597-026-07183-6 (PMC13243636; doi:10.1038/s41597-026-07183-6)
Supplement: Supplementary file 2 — Supplementary information [file 41597_2026_7183_MOESM2_ESM.pdf]

nature portfolio

Corresponding author(s): P. Ghaffarzadeh (p.ghaffarzadeh@hull.ac.uk)
Last updated by author(s): 12-03-2026

Machine Learning Checklist V 1.1

Nature Portfolio wishes to improve the reproducibility of the work that we publish. This form is intended to provide structure for consistency and transparency in reporting of works using or developing Machine Learning models. Some list items might not apply to an individual manuscript, but all fields must be completed for clarity.

1. Availability and reproducibility of Code and Data

Please select all that apply regarding the availability of the data and code used in the study.

- ☐ Code will be included in a CodeOcean capsule.
- ☑ The source code is included in the submission or available in a public repository:
  https://github.com/ParvinGhaffarzadeh/Apple-Watches-and-Force-Plate-dataset
- ☐ A compiled standalone version of the software is included in the submission or available in a public repository:
  N/A
- ☑ A test dataset and instructions/scripts for replicating the results are included in the submission or available in a public repository:
  https://doi.org/10.5281/zenodo.17376717
- ☑ A Readme file with instructions for installing and running the code is included in the submission or available in a public repository:
  https://github.com/ParvinGhaffarzadeh/Apple-Watches-and-Force-Plate-dataset
- ☑ The code is made available to reviewers during review.
- ☐ Pretrained models are used in the study and accessible through: N/A
- ☐ Pretrained models are used in the study and are not accessible: N/A
- ☑ The paper contains information on how to obtain code and data after publication.

2. Datasets

A. All data sources are listed in the paper.

- ☑ Yes
- ☐ No

B. The train, test and validation datasets are publicly available, and links/accession numbers have been provided in the manuscript or supplementary materials.

- ☑ Yes
- ☐ No

C. We have reported and discussed potential dataset biases in the paper. Where applicable, appropriate mitigation strategies were used.

☑ Yes — Usage Notes and Limitations sections; small sample (n=10), healthy adults aged 26–41, controlled lab conditions; single manufacturer (Apple Watch); generalisability to clinical or elderly populations requires further validation.

☐ No

D. The data cleaning and preprocessing steps are clearly and fully described, either in text or as a code pipeline.

☑ Yes — Methods: Data Processing, Quality Control, and Temporal Alignment sections; preprocessing scripts archived on Zenodo and GitHub.

☐ No

E. Instances of combining data from multiple sources are clearly identified, and potential issues mitigated.

☑ Yes — Trial Matching and Manifests section; temporal alignment of wrist IMU, waist IMU, and force plate described in detail; hybrid alignment pipeline with cross-correlation validation applied.

☐ No

3. Model and training

A. What model architecture is the current model based on? N/A — This is a Data Descriptor; no ML model is developed or trained in this paper.

B. A Model Card is provided.

☐ Yes

☑ No — This is a Data Descriptor; no ML model is developed or trained.

C. The model clearly splits data into different sets for training (model selection), validation (hyperparameter optimization), and testing (final evaluation).

☐ Yes

☑ No — This is a Data Descriptor; no ML model is developed or trained.

D. The method of data splitting (e.g. random, cluster- or time-based splitting, forward cross-validation) is clearly stated.

☑ Yes — Usage Notes: ML considerations; participant-stratified splits (Leave-One-Participant-Out / GroupKFold) explicitly recommended. Random row-wise splits explicitly prohibited due to temporal autocorrelation.

☐ No

E. The data splitting mimics anticipated real-world applications.

☑ Yes — Leave-One-Participant-Out cross-validation recommended to reflect generalisation to unseen individuals, consistent with real-world deployment.

☐ No

F. The data splitting procedure has been chosen to avoid data leakage.

☑ Yes — Recommended ML Usage Checklist (Usage Notes): random row-wise splits explicitly prohibited because rows within a trial are temporally autocorrelated and share participant identity, causing severe data leakage.

☐ No

G. The interpretability of the model has been studied and clearly validated.

☐ Yes

☑ No — This is a Data Descriptor; no ML model is developed or trained. Interpretability is not applicable.

4. Evaluation

A. The performance metrics used are described and justified in the paper.

☑ Yes — Technical Validation: ICC (two-way mixed, absolute agreement), CV%, and Pearson r reported and justified. Pearson r explicitly framed as a plausibility and QC metric only, not a validity measure.

☐ No

B. Cross-validation of the results is included.

☐ Yes

☑ No - This is a Data Descriptor; no ML model is trained or evaluated. Cross-validation is not applicable. Repeatability is assessed via ICC across repeated trials (Table 5).

C. Community-accepted benchmark datasets/tasks are used for comparisons.

☑ Yes — Table 1: systematic comparison with Winter (1983), Fukuchi et al. (2017, 2018), Camargo et al. (2021), Scherpereel et al. (2023), and other established open-source biomechanics datasets.

☐ No

D. Baseline comparisons to simple/trivial models (for example, 1-nearest neighbour, random forest, most frequent class) are provided.

☐ Yes

☑ No — This is a Data Descriptor; no ML model trained or evaluated. Baseline model comparisons are not applicable.

E. Benchmarks with current state-of-the-art are provided.

☐ Yes

☑ No — This is a Data Descriptor; no ML model trained. The dataset is released to enable future benchmarking by the community.

F. Ablation experiments are included.

☐ Yes

☑ No — This is a Data Descriptor; no ML model developed. Ablation experiments are not applicable.

G. The model has been tested on a fully independent dataset.

☐ Yes
☑ No — This is a Data Descriptor; no ML model trained or evaluated.

5. Computational resources

A. The paper contains information on hardware/computing resources that were used.

☑ Yes – Apple Watch Series 5+ (accelerometer ±8g, gyroscope ±2000°/s, ≈100 Hz) and AMTI OR6-7 force plate (1000 Hz) are described in the Sensor Specification section.

☐ No

B. The paper includes information on the computational costs in terms of computation time, parallelization or carbon footprints estimates.

☐ Yes
☑ No — This is a Data Descriptor. Computational cost reporting is not applicable.
